# Health Minister's Statement

**Published:** 1920-07-24

**Authors:** 


THE PUBLIC WELL-BEING—(*continued*).

### Health Minister's Statement.

### Valuable Points of Progress.

The debate in Parliament on the Health Services Vote was a singularly unattractive affair. The Minister of Health (Dr. Addison) is not an inspiring orator, but, though his speech lacked inspiration, it contained a steady and straightforward account of progress and development. What was more disappointing was the dull and uninteresting level of the discussion, which showed scarcely a sign of enlightenment on the vital question of public health.

Dr. Addison wisely declared that a sound health policy would require time and patience for its development, and the first essential was the spread of good common information. All schemes must be designed with the dominant intention of prevention, and it was in this respect that we had not made such progress as we might have done; but it was not possible to produce trained staffs in a year.

Above all, we must have at our service at all times an active prosecution of scientific research. The preventive services we had developed in this country had up to the present, so far as they

THE PUBLIC WELL-BEING—*(continued)*

related to the surroundings of individuals, reached a stage of development beyond that of any other. Our sanitary services were well developed, and it was largely owing to them that the health of our people, who in many cases dwelt in most pestilential places, was as good as it was. In so far as the Ministry of Health was really successful, and as, in course of time, its services became more complete and yielded a better result, the less obvious and the less striking they would be.

Progress in housing gave no additional information likely to excite much enthusiasm, but there is a steady, if slow, improvement. The record of the steps taken to prevent the importation of disease into the country was one of foresight and efficiency; there was a promising account of the experience of village colonies so far established for consumptives; and the use made of centres of treatment for venereal disease shows that their value is realised (they were utilised by 843,000 new patients: this is some indication of the distressing extent of venereal diseases).

Some of the most encouraging points were those affecting mothers and children. More than 700 women are being prepared under training schemes for service as midwives, and Dr. Addison was justified in describing as startling the figures which he gave disclosing the recent improvement in the infant death-rate. Towards the end of last century and at the beginning of this the rate was practically stationary at about 150. It began to fall in 1906, when it was 132; in 1918 it was 97; and last year 89. For the last three quarters of last year and the first quarter of this the figure was only 78—about half what it was twenty years ago. If that had been the rate during the last ten years it would have meant a saving of 210,000 lives in the aggregate The improvement to be expected among children who did not die has not yet made itself sufficiently felt in the schools. Of the first 170,000 children going to the schools who were examined last year 49 per cent. were found to be physically defective, but the figures will drop as the improvement going on makes itself felt later.

The Minister's references to the voluntary hospitals seemed to indicate that the Government had no present intention of interfering, but are leaving the King Edward Fund to meet immediate necessities, though Dr. Addison did say—whatever this may imply—that other proposals are being worked out. On the subject of research he rightly pointed to the remarkable differences of typhus mortality in the South African war and the last war as an overwhelming justification for expenditure on intelligently directed research, and it is a welcome announcement that the Ministry is making further provision for scientific and medical research.

Apparently the only member who in the subsequent debate attempted to deal with health as apart from housing was Dr. Raw, of Liverpool. He gave his blessing to the village colonies for consumptives, hoped the Ministry would deal with the difficult question of tuberculous milk, wanted to see the general transformation of Poor-Law infirmaries into municipal hospitals—an experiment begun in Bradford—congratulated the Ministry in its efforts to get more for research, and declared that the Minister's speech fully justified the formation of the Ministry.

